# An integrated approach for rare disease detection and classification in Spanish pediatric medical reports

**DOI:** 10.1038/s41598-025-21827-4

**Published:** 2025-10-30

**Authors:** Andres Duque, Lourdes Araujo, Juan Martinez-Romo, María D. Esteban-Vasallo, María-Felicitas Domínguez-Berjón, David Malillos Perez

**Affiliations:** 1 Departamento de Lenguajes y Sistemas Informáticos, ETS Ingeniería Informática, Universidad Nacional de Educación a Distancia (UNED), 28040 Madrid, Spain; 2 Instituto Mixto de Investigación, Escuela Nacional de Sanidad (IMIENS), 28029 Madrid, Spain; 3 Dirección General de Salud Pública, Consejería de Sanidad de la Comunidad de Madrid, 28002 Madrid, Spain; 4 Servicio Madrileño de Salud, Dirección Técnica de Sistemas de Información, Gerencia Asistencial de Atención Primaria, 28035 Madrid, Spain

**Keywords:** Rare disease detection, Natural language processing, Spanish medical reports, Large language models, Keyphrase-based information extraction, Computer science, Scientific data, Diseases, Disease prevention, Public health

## Abstract

Rare disease detection and classification is one of the most significant challenges in the application of Natural Language Processing techniques to the analysis and extraction of information from biomedical texts. In this paper, we present a novel research focused on the detection and classification of rare diseases in clinical notes extracted from a cohort of pediatric patients from the Community of Madrid in Spain. From a set of collected and anonymized medical records, we propose a semi-supervised, keyphrase-based system to perform an initial detection of mentions of rare diseases, which is then validated and refined by experts to build a consolidated dataset concerning a subset of different rare diseases. Based on this dataset, we carry out a series of experiments for rare disease classification using both a semi-supervised technique and state-of-the-art supervised systems based on both discriminative and generative models. A detailed case analysis provides insights on which systems excel in specific scenarios and why. The validated dataset contains a total of 1900 annotated texts containing mentions to rare diseases. Experiments on this dataset show that the best supervised models improve the performance of the semi-supervised system by more than 10% (78.74% vs 67.37% micro-average F-Measure), individually enhancing the classification of a significant number of diseases in the dataset. State-of-the-art supervised systems are able to offer promising results on the detection and classification of rare diseases in clinical texts, even in cases for which the amount of annotated information is low. On the other hand, semi-supervised models present interesting capabilities for dealing with limited information and data in the field.

## Introduction

Rare diseases (RDs) are defined by their low prevalence, with varying criteria worldwide: under 200,000 cases in the U.S. (60 per 100,000), $\le$ 50 per 100,000 in the EU, and < 65 per 100,000 by WHO standards. Despite their low prevalence, the broad spectrum of rare diseases results in a high number of affected individuals. According to various organizations focused on rare diseases, over 300 million people worldwide live with a rare disease. In other words, these conditions affect approximately 3.5% to 5.9% of the global population ^1^.

Rare diseases profoundly affect individuals and families emotionally, economically, and socially. Their chronic nature and uncertain treatment often cause psychological distress and a reduced quality of life. High medical costs and limited diagnostic access, compounded by physician knowledge gaps and patient dispersion, delay diagnosis. The challenge of diagnosing rare diseases arises from their low prevalence, limiting case studies and making pattern recognition difficult^2^. General physicians play a key role in guiding diagnosis, but with 6,000–8,000 rare diseases, comprehensive knowledge is unfeasible.

In particular, rare diseases bring unique challenges for young people, disrupting their development, education, social integration and opportunities for the future. Many rare diseases cause disabilities, often related to congenital malformations: structural or functional abnormalities, such as metabolic disorders or hearing defects, identifiable before or after birth. Disabilities often exacerbate the rare diseases problems, so early intervention and strong support systems are essential to minimize long-term effects. Orphanet (https://www.orpha.net/), the international database and portal dedicated to rare diseases and orphan drugs, collects data to improve understanding^3^, raise awareness and provide tools to affected individuals. Advanced techniques, such as deep learning, can help uncover connections between these malformations and rare diseases, leading to improved characterization and identification of these conditions.

Another frequent feature in the mentions of rare diseases is the inconsistency of their naming, which creates confusion, complicates data retrieval, and hinders registry maintenance. For instance, conditions like Ehlers-Danlos syndrome may be listed under different terms, such as “cutis laxa” or “joint hypermobility syndrome”, making case tracking and research more challenging.

For the above reasons, centralized registries are vital for improving rare diseases diagnosis and research. They address challenges like low prevalence, varied naming, and lack of specific medical codes, consolidating data for accurate diagnosis, treatment, and better patient outcomes.

The Regional Registry of Rare Diseases (SIERMA) aims to improve rare disease detection in the Community of Madrid by integrating data from various healthcare systems^4^. Managed by the Directorate General of Public Health, it collects information mainly from electronic health records (EHRs) in primary care, but also hospital discharge data, mortality records, and more. EHRs in Primary Care use the ICPC-2 (International Classification of Primary Care) coding system, which facilitates coding for common conditions but lacks specific codes for many rare diseases.

In this context, Natural Language Processing (NLP) methods offer significant potential to enhance the identification of diagnoses such as rare diseases through the analysis of textual information from unstructured clinical texts. Given the scarcity of annotated data and the linguistic variability inherent in clinical narratives, supervised approaches often face limitations due to the high cost and complexity of data labeling. Hence, semi-supervised or knowledge-based methods, such as pattern-matching techniques leveraging domain-specific keyphrases and external knowledge sources, can provide valuable initial screening tools that do not rely on extensive labeled datasets. These methods can capture relevant clinical concepts and linguistic patterns indicative of rare diseases, enabling the extraction of meaningful information even in low-resource scenarios. By combining semi-supervised keyphrase-based approaches with advanced transformer-based models, this work aims to leverage the strengths of both strategies, providing robust and scalable solutions that can assist clinicians and researchers in improving rare disease diagnosis and documentation.

## Background

Efforts have been made to detect mentions of disabilities in texts^5^, as well as their relationship with rare diseases^6^, applying NLP techniques. By employing NLP, researchers aim to extract and analyze textual data from various sources, such as electronic health records and medical literature, to identify references to disabilities and their association with rare diseases. These techniques enable the automated processing of vast amounts of text, allowing for the identification of patterns and insights that can aid in understanding the impact of rare diseases on individuals’ abilities and overall health. Moreover, leveraging NLP facilitates the development of computational tools and algorithms for more efficient and accurate detection of these associations, ultimately contributing to improved diagnosis, treatment, and management of rare diseases and associated disabilities.

In the absence of training data, some rule-based systems have been proposed^7^. Dong et al.^8^ have developed a system relying on weak supervision. This system links rare diseases names (Orphanet Rare Disease Ontology (ORDO)) to UMLS codes. They create training data of candidate mention-UMLS pairs by applying some custom rules. The linked pairs created in this way are then used to train a model. However, the experiments have been carried out on a general medical dataset, MIMIC-III^9^, not specific for rare diseases.

Early rare disease detection systems, when equipped with reference data, initially relied on classical classifiers^10^, followed by advancements in deep learning systems^6,11^. More recently, with the emergence of Large Language Models (LLMs), efforts have begun to explore options for applying them to this problem. LLMs offer promising capabilities in processing and understanding large volumes of textual data, potentially enhancing the accuracy and efficiency of rare disease detection by leveraging their advanced NLP capabilities.

Wang et al.^12^ have developed a method for rare disease concept normalization by fine-tuning Llama 2, an open-source LLM using a domain-specific corpus sourced from the Human Phenotype Ontology (HPO). They used a template-based script to generate two corpora for tuning. The first (NAME) contains normalized HPO names, extracted from HPO vocabularies, along with their corresponding identifiers. The second (NAME+SYN) includes HPO names and half of the synonyms of the concept, as well as their identifiers. The fine-tuned models show ability to standardize phenotype terms not encountered in the fine-tuning corpus, encompassing misspellings, synonyms, and terms sourced from alternate ontologies.

Oniani et al.^13^ propose a majority voting system that combines several LLM systems on one-shot rare disease identification and classification tasks. Few-Shot Learning (FSL) is a subfield of artificial intelligence aimed at enabling machine learning even in scenarios with a limited number of samples (also referred to as “shots”). Identifying rare diseases stands out as a natural application for leveraging FSL techniques. The system works by prompting (giving instructions or questions to an AI model to get an answer or result) several LLMs to perform the same task and then conducting a majority vote on the resulting outputs. The ensemble method improved the results of all the individual models. The proposal was evaluated on a novel Few-Shot Learning (FSL) dataset for rare disease identification, obtained by processing a recently published MIMIC-IV database^14^.

Although the amount of data available in the field of rare diseases is very scarce, some exceptions can be found. One of them is the RDD (Rare Disease-Disabilities) corpus^6^. It is composed of scientific abstracts of articles in English related to some rare diseases. The annotation includes disabilities, negation, speculation and also relationships between rare diseases and disabilities. In addition to providing annotations of mentions to rare diseases, it has annotated disabilities using the Orphanet Functioning Thesaurus as the base of the annotation criteria. Another corpus related to the previous one is DIANN^15^, which does not focus on rare diseases but on disabilities. It is also made up of abstracts of scientific articles, but in this case in English and Spanish. The RareDis corpus^16^ is compiled from texts extracted from the rare disease repository, curated and updated by the National Organization for Rare Diseases (NORD). It includes annotations of different entities (disease, rare disease, symptom, sign and anaphor) as well as some relationships (produces, is a, is acronym, is synonym, increases risk of, anaphora). The Boston Children’s Hospital^17^ is developing the Children’s Rare Disease Cohorts initiative (CRDC), a database of 2441 exomes from 15 pediatric rare disease cohorts, with major contributions from early onset epilepsy and early onset inflammatory bowel disease. All sequencing data are integrated and combined with phenotypic and research data in a genomics learning system. Phenotypes were both manually annotated and pulled automatically from patient medical records. Kariampuzha et al.^18^ have also compiled a corpus of scientific articles, in this case with annotations of epidemiological information (epidemiologic type, epidemiologic rate, location, ethnicity/nationality/race, date, sex, disease name and synonym, and disease abbreviation). They randomly selected 500 rare diseases and then they selected a maximum of 50 PubMed abstracts for each disease.

Existing corpora publicly available are often pieced together from scientific articles or clinical case studies, lacking comprehensive medical reports essential for in-depth analysis and model training for this type of documents. In this work we focus precisely on primary care medical reports, developing systems specifically designed for this context.

### Current limitations and proposal

Despite the progress made, several reviews of the literature on the subject^19,20^ have concluded that current technology encounters significant difficulties in its application to the study of rare diseases, and that information sharing is essential.

Another problem is that most existing works in this domain still rely significantly on manual effort, with minimal automation, making the process of detecting rare diseases time-consuming and prone to human error. A major challenge is the diversity in nomenclature, as rare diseases often have multiple names or aliases, leading to inconsistencies in recognition. Furthermore, the complexity of medical language adds to the difficulty, as systems must handle nuances such as negations (e.g., “no evidence of disease”) and references to diseases affecting family members rather than the patient. Most critically, the lack of sufficient data on rare diseases severely hampers the development and training of robust, automated extraction tools, making it difficult to achieve reliable performance. In the field of rare diseases, the availability of openly accessible corpora remains notably sparse, notwithstanding the exceptions mentioned above, a deficiency that interferes with the advancement of research in this critical domain.

We aim to address these limitations to enhance the effectiveness of the rare disease registry in a densely populated area of Spain, specifically in the Community of Madrid.

For this purpose, a study has been conducted to determine the types of systems most suitable for working with the available data, this is, a limited number of cases for each rare disease and records written in Spanish. Among the latter, the most recent Transformer-based models including open-source generative models have been studied.

Not only have we evaluated both types of models, but we have also evidenced that the combination of both types of techniques can lead to high accuracy results with limited manual annotation effort. The thorough identification of new rare disease cases that have not been previously detected not only represents a significant improvement for the healthcare system, which can then provide support to patients affected by these diseases, but also increases the amount of available information about these diseases, which is crucial for research given their nature.

In addition, the outcome of the study is a system designed to process new EHRs as they become available, with the ability to identify a certain set of rare diseases, which may not have been adequately recorded. For the evaluation, we have focused on a set of rare diseases of special interest in the registry of rare diseases in the Spanish state registry.

## Materials and methods

### Rare disease detection

This section is dedicated to describing the pipeline followed for building a dataset composed of texts mentioning rare diseases. First, we depict the original source of information from which the textual data is extracted for its use throughout this research. Based on this data, a semi-supervised knowledge-based system for extracting mentions of rare diseases is developed, being its results subsequently validated by experts in the field. Once this validation is generated, the information is used to finally develop a consolidated dataset for further experimentation aimed at the detection and classification of rare diseases.

**Fig. 1:**
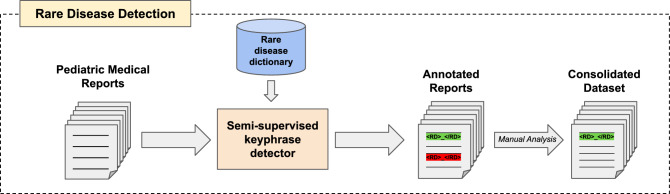
Rare disease detection pipeline.

Figure 1 illustrates the pipeline for detecting rare diseases in clinical notes using the proposed semi-supervised system, followed by the manual validation done by experts in the field.

#### Data cohort

The initial data cohort used in this research is a collection of clinical reports written in the Spanish language related to pediatric consultations in Primary Care Centers of the Community of Madrid. The study population consisted in all children born between 01/01/2010 and 08/25/2021 with access to the National Health Service in the Community of Madrid. All the information registered in the electronic clinical records of primary care under a ICPC-2 code corresponding to a congenital malformation was obtained for this population. A process of anonymization and obfuscation was carried out on these data, to avoid any possibility of identification. Data were anonymized and obfuscated by professionals from the Regional Registry of Rare Diseases. For anonymization, each individual was assigned an automatically generated numerical identifier, and all personal identification data (such as first and family names, medical record numbers, etc.) were removed from the final dataset. Any potentially sensitive information within the clinical notes was also obfuscated in the final dataset. To achieve this, a comprehensive dictionary of nearly 550,000 terms and text sequences was used. This dictionary was compiled from population databases in our region (including census data) and includes first and family names, geographical names (cities, municipalities, districts, neighborhoods), and names of hospitals and health centers. To prevent accidental removal of rare disease names or syndromes, the dictionary was cross-checked against the Orphanet rare disease catalogue to exclude any overlapping terms. The obfuscation process automatically scans the clinical notes for matches against this dictionary, replacing any detected term with a sequence of asterisks of equal length to maintain text readability. Additionally, sequences of three-digit numbers (e.g., phone numbers or dates) are replaced to protect other sensitive information. The entire process is supervised and evaluated by public health professionals, who also manually enrich the dictionary to ensure its accuracy and effectiveness. In this sense, it is important to remark that the research project has been approved by the regional Research Ethics Committee for Medicines (“Comité de ética de la Investigación con medicamentos” or CEIm) of the Community of Madrid^21^. This approval confirms that the study and all methods involved in it comply with the relevant guidelines as well as with national and European data protection laws and ethical regulations. In this context, and as confirmed by the ethics committee, informed consent from individual participants is not required due to the full anonymization of the data and the observational nature of the study.

Each analyzed record contains an anonymized identifier for the patient, as well as an identifier for each specific episode representing a set of visits to the pediatrician for monitoring a medical issue. Each episode may be divided into different notes indicating each visit to the pediatrician, and the date of each note is also recorded. Each of these notes, therefore, contains the text written by the health professional during the visit. It is this textual information that will be used for the process of searching for mentions of rare diseases. It is important to highlight that the selected pediatric consultations are associated with the study of malformations and congenital anomalies in patients. That is, all medical histories within the analyzed corpus refer to follow-ups related to a congenital anomaly or malformation. These malformations are coded using the aforementioned ICPC-2 codes. The specific ICPC-2 codes that can be found in the data cohort, together with their Spanish and English descriptions, are shown in Appendix A (Table A1).

The initial dataset we are working with consists of a total of 249,950 clinical notes, belonging to 86,343 patients during the follow-up of 96,158 episodes. That is, each patient has an average of 1.11 consultation episodes, and each episode consists of an average of 2.60 consultations, each containing a clinical note taken by the health professional.

#### Keyphrase-based semi-supervised rare disease detection

Considering that the original dataset consists of raw texts without any type of annotation whatsoever regarding rare diseases, the need to develop a semi-supervised system to perform an initial detection of mentions of these entities is clear. This system must therefore be capable of locating possible text fragments that allude to the presence of a rare disease in the patient’s medical history. This section describes the developed system, which makes use of a dictionary of rare diseases and their naming variants, combined with a series of linguistic rules that allow us to detect rare diseases in the original text. As shown in the following sections, the proposed system is based on a pattern-matching pipeline in which keyphrase extraction is complemented by a series of processing steps specifically designed for the task at hand. These steps include pre-processing, filtering of candidate keyphrases using TF-IDF, and post-processing rules based on the detection of negation and references to individuals other than the patient (specifically, family members).

Rare disease dictionary The dictionary of rare diseases that are searched for within the texts has been generated from the public information provided by Orphanet. Specifically, a database of rare diseases identified by unique codes, denoted ORPHA codes, is provided. Mentions of rare diseases can appear in biomedical texts in various ways, so Orphanet makes a great effort to include all variants and synonyms related to a rare disease in this database. Through the automatic processing of the database, we are able to generate a dictionary that contains a total of 9,317 different diseases, with an average of 2.24 different variants per rare disease. Therefore, the dictionary is populated with a total of 20,894 potential expressions to be found within the clinical reports. Some examples of the elements populating the dictionary can be seen in Table 1:

**Table 1:** Examples of rare diseases and their variants extracted from the Orphanet database. Italic font is used after each Spanish expression for indicating the English translation (in parentheses).

ORPHA code	Rare disease	Variants
249	Displasia fibrosa de hueso *Fibrous dysplasia of bone*	Displasia ósea fibrosa (*Fibrous bone dysplasia)*
666	Osteogénesis imperfecta *Osteogenesis imperfecta*	Enfermedad de Lobstein (*Lobstein disease)* Enfermedad de Porak y Durante (*Porak and Durante disease)* Enfermedad de los huesos de cristal (*Brittle bone disease)* OI (*OI)* Osteopsatirosis (*Osteopsathyrosis)*
886	Síndrome de Usher *Usher’s Syndrome*	Retinosis pigmentaria-sordera (*Retinitis pigmentosa-deafness)* USH (*USH)*
79253	Fenilcetonuria leve *Mild phenylketonuria*	PKU leve (*Mild PKU)* Variante de la PKU (*PKU variant)* Variante de la fenilcetonuria (*Phenylketonuria variant)* mPKU (*mPKU)*

As it can be observed in the table, there are different types of variants regarding a particular rare disease, such as acronyms (“*OI*” for “Osteogénesis imperfecta”), linguistic variations (“*Displasia ósea fibrosa*” instead of “*Displasia fibrosa de hueso*”), completely different ways of naming a disease (“*Retinosis pigmentaria-sordera”* for “*Síndrome de Usher*”) or even acronyms derived from the English name of the disease (“*PKU*” for “*Fenilcetonuria*”, coming from “Phenylketonuria”). This enormous variability regarding the ways of naming rare diseases in the literature is one of the most crucial aspects for developing a system able to accurately detect rare diseases within medical texts.

Keyphrase extraction In addition to generating the dictionary of rare diseases and their possible variants, a specific processing of the texts in with mentions of these diseases will be searched is needed. The most important operation performed on these texts is the extraction of keyphrases that can represent the most important information mentioned in the text. This greatly reduces the complexity of conducting an exhaustive search for all possible rare diseases across all texts.

A basic pre-processing is performed on the original texts to prepare them for keyphrase extraction. This pre-processing consists of basic tokenization including sentence and word splitting using the NLTK Python library^22^, removal of punctuation elements, lowercasing, and removal of accents. Additionally, the lemmatization of the words in the text allows us to reduce lexical variability for better subsequent detection. At the same time, POS-tagging of the text is performed using the TreeTagger tool^23^ in order to obtain the grammatical categories of the words, which will be used in the keyphrase extraction.

Once the texts have been prepared, a regular expression regarding POS tags of the words in the text is proposed for extracting the keyphrases of interest:

(NEG? JJ* (NN .*) $^+$ JJ* IN)? JJ* (NN.*) $^+$ JJ*

In this expression, “NEG” indicates negation triggers in Spanish such as “*no*” or “*sin*”, “JJ” represents adjectives, “NN” is the tag for nouns and “IN” marks the apparition of a preposition. This regular expression mainly describes the occurrence of a single noun or a noun phrase (last three POS tags), that can or cannot be accompanied by a prepositional phrase. A rare disease is normally expressed through a noun or a noun phrase (for instance, “*Fenilcetonuria leve*” or “*Osteogénesis imperfecta*”). However, there are cases in which rare disease are named using more complex sentences containing single and compound prepositional phrases (i.e., “*Síndrome de Usher*”, “*Enfermedad de Lobstein*” or “*Inmunodeficiencia por deficiencia de factor H*”). The complete regular expression might be useful for detecting those particular cases. This strategy has shown to be useful on extracting relevant keyphrases from biomedical texts in previous works^24^.

Through this expression, only the keyphrases of interest are selected, eliminating the rest of the textual content. Once this process is completed, a TF-IDF model is constructed in which each document is the text of an entry from the original dataset, containing a note about the patient’s medical history, and the elements or tokens of the documents are the extracted keyphrases. The TF-IDF model allows us to further reduce the system’s complexity by focusing on the most relevant keyphrases for each document. Specifically, we select a maximum of 50 keyphrases for each note in the corpus. The combination of using regular expressions for keyphrase extraction along with the TF-IDF model to select the most relevant keyphrases enables filtering out potential expressions that could introduce noise or ambiguity in the detection of rare diseases. Therefore, this provides an advantage over other dictionary-based systems aimed to detecting expressions through rule-based pattern matching, such as the SpaCy tool (https://spacy.io/).

The same pre-processing steps (tokenization, punctuation and accent removal, lowercasing and lemmatization) is performed on the names of the rare diseases and their variants in the dictionary. In addition, both the keyphrases extracted from the text and the names and variants of the rare diseases are transformed into bags of words, where the order of the words is not representative. This way, the system is able to deal with the high variability regarding word order in the Spanish language, in which expressions such as “*Condromalacia rotuliana familiar*” and “*Condromalacia familiar rotuliana*” would be equivalent.

The final step is the exhaustive search for matching expressions of rare diseases and their variants throughout all the extracted keyphrases in the considered texts. For this purpose, the set of keyphrases belonging to a particular document (clinical note), converted into bags of words to disregard the word order, is compared with the complete set of rare diseases, also converted into bags of words. The matches found are stored as rare diseases mentioned in a clinical note.

A post-processing step is also applied using some rules to reduce ambiguity and noise. In particular, detected diseases composed of only one word are disregarded if the word has three or fewer letters. In such cases, there is a high probability of having detected an acronym, and empirical evidence shows that the error rate increases significantly. Similarly, if the detected disease consists of a single word and that word is “syndrome”, the detection is also discarded due to its considerable ambiguity.

Post-processing: negation and family There are two fundamental cases in the proposed pipeline that are crucial to consider in order to avoid errors while detecting mentions of rare diseases. On one hand, it is important to consider negation, that is, mentions of rare diseases that, even though they appear in the text, do not indicate that the patient suffers from them. These are usually cases where, after the suspicion of a certain disease, it has been ruled out after conducting tests.

On the other hand, a similar consideration must be made regarding mentions of rare diseases related to the patient’s family, that is, cases where a family member of the patient has a certain rare disease mentioned in the text, but this disease does not refer to the patient attending the health professional.

In order to address negation cases, a subsystem based on the NegEx algorithm^25^, adapted to the Spanish language, has been employed. After a collection of triggers regarding negation is defined, the algorithm performs a rule-based analysis of a given text and a particular entity (in our case, the rare disease), to determine whether the entity appears negated in the text. The same idea is applied for detecting mentions related to family members, however, in this case, the collection of triggers involves words and expressions that refer to members of the family. In particular, four different types of triggers can be defined in our case within the NegEx algorithm: pre-negations ([PREN] tag) for triggers commonly appearing previous to the negated entity (for instance, “*sin evidencia*”, “without evidence”), post-negations ([POST] tag) for triggers commonly appearing after the negated entity (for instance, “*debe ser descartado*”, “must be discarded”), pseudo-negations ([PSEU] tag) for expressions that introduce some kind of doubt regarding the occurrence or not of the entity (for instance, “*podría ser descartado*”, “could be discarded”), and conjunctions ([CONJ] tag) for expressions that might allow to determine the degree of uncertainty for negating a particular entity (for instance, “*secundario a*”, “secondary to”). On the other hand, family triggers only make use of previously appearing triggers and conjunctions. Once these triggers have been determined, the NegEx algorithm receives the entity of interest (in our case, a particular rare disease), and the text (clinical note) within possible negations or family mentions should be searched related to that entity. The algorithm then returns whether the entity is considered to be negated, or related to a family member, within the text.

Tables 2 and 3 show examples of negated mentions of rare diseases, and rare diseases related to family members, respectively.

**Table 2:** Examples of negated mentions of rare diseases.

Rare disease	Negated mention	Negated mention (EN)
Leucomalacia periventricular	No se observan signos de hemorragia antigua de la matriz germinal **ni leucomalacia periventricular**	***No signs of **** old hemorrhage in the germinal matrix or* *** periventricular leukomalacia**** are observed*
Craneosinostosis	RX de cráneo realizada por neurocirugía que según refiere **no hay signos de craneosinostosis**	*Skull X-ray performed by neurosurgery, which reportedly shows* ***no signs of craniosynostosis***
Enfermedad neuromuscular	Cuádriceps no presenta degeneración grasa por lo que **no debe corresponder a una enfermedad neuromuscular**	*Quadriceps not showing fatty degeneration, so* ***it should not correspond to a neuromuscular disease***

**Table 3:** Examples of mentions of rare diseases affecting family members.

Rare disease	Family mention	Family mention (EN)
Enfermedad de Charcot	**Padre** nacido en ****, **con enfermedad de Charcot**	***Father*** born in ****, with ***Charcot’s Disease***
Malformación aórtica	Acude la madre refiriendo que han intervenido al **padre** de una **malformacion aortica**	*The mother reports that the* ***father**** has undergone surgery for an* ***aortic malformation***
Miocardiopatía hipertrófica	**Abuelo** diagnosticado de **miocardiopatia hipertrofica**	***Grandfather**** diagnosed with* ***hypertrophic cardiomyopathy***

Once that these mentions have been detected within a text, these texts are annotated with one out of three different labels regarding negation: “affirmed”, “negated” and “possible”. This last possible annotation is employed in cases for which the same rare disease appears both affirmed and negated in the analyzed text. Regarding family, and following the same considerations, three different labels can be assigned to a text: “individual” for rare diseases related to the patient, “family” for rare diseases referring to family members, and “individual and family”. The last case is employed when a rare disease appears both related to the patient and to a family member in the same text.

It is important to remark that the post-processing steps that involve detection of negated rare diseases and mentions to family members are conducted prior to the manual annotation and validation of the dataset by the experts, which will be detailed in “Dataset creation”. This is, information regarding negation and family history is added to the automatically annotated dataset aiming to guide human annotators in the process of confirming or rejecting the mention of a rare disease within a clinical note. The final decision to validate the appearance of a rare disease within the text always rests with the human annotators of the consolidated dataset.

#### Dataset creation

The output of the whole process described in “Keyphrase-based semi-supervised rare disease detection” is a subset of annotated clinical notes, each of them related to a particular consultation within an episode in the patient’s medical history, for which at least one mention of a rare disease has been found in the text. Moreover, the previously mentioned annotations regarding negation and family-related mentions are also included in this output.

Considering that this is the output of an automatic system, and we are working in the medical domain with sensitive data, there is an important need of contrasting this information with experts that can validate it. Hence, this is the data collection from which the final rare disease dataset will be built, after careful examination and validation by these experts. The main statistics of this initial resource are gathered in Table 4.

**Table 4:** Statistics of the initial collection after applying the semi-supervised detection of rare diseases. “RDs” stands for “Rare diseases”, while “I & F” stands for “Individual and Family”.

No. notes	No. patients	No. RDs	Negation			Family		
			Affirmed	Negated	Possible	Individual	Family	I & F
9,885	6,188	294	8,947	802	136	9,502	298	85

As shown in the table, there are a total of 9,885 clinical notes that contain mentions of rare diseases, for a total of 6,188 unique patients. The vast majority of mentions are affirmed and refer to the individual, although negation and mentions of family members are phenomena that occur frequently, making it important to consider them. On the other hand, a total of 294 different rare diseases are detected.

Regarding the potential overlapping between detected entities in the initial collection, it is important to remark that in the first automatic annotation, there will be a higher overlap between clinical entities with different assigned Orpha codes. This is due to the hierarchical classification of rare diseases considered by Orphanet, in which two clinical entities referring to the same disease but with different levels of granularity, may present different Orpha codes. Table 5 gathers this information about the co-occurrence of different clinical entities (potential rare diseases) in the initial collection, after applying the keyphrase-based detection technique. As it can be observed in the table, most of the clinical notes and patients only present one clinical entity, however, there are a significant number of cases (both clinical notes and patients) for which the appearance of two different clinical entities is quite common. On the other hand, the co-occurrence of three or more clinical entities is not so usual. It is important to remark that in this case, 294 different clinical entities with their corresponding Orpha codes are initially detected as rare diseases. Hence, and as a result of the granularity issues regarding the hierarchy in Orphanet previously mentioned, it is possible that a pair of co-occurring clinical entities are similar or highly related although classified differently according to the rare disease dictionary employed for their detection. For instance, “hipotiroidismo congénito” (congenital hypothyroidism, Orpha code 442) and “hipotiroidismo primario congénito” (primary congenital hypothyroidism, Orpha code 226295) have been annotated as the same disease for cases included in the final, consolidated dataset.

**Table 5:** Clinical entity overlapping in the initial collection. Column “Notes” refers to clinical notes, while column “Patients” refers to unique patients in the cohort.

Different clinical entities	Notes	Patients
1	8,540	5,402
2	594	647
3	47	109
> 3	4	30
Total	9,886	6,188

From this initial collection, a verification and validation process is carried out by expert personnel from the SIERMA group of the Community of Madrid. In particular, four healthcare professionals (medicine, nursing, and speech therapy) with several years of experience participated in the review and validation of the original annotation. Case validation is performed by a detailed review of the complete electronic clinical records of the individual (primary care and hospital consultations, and hospital admissions), confirming or rejecting the mentions of a specific subset of rare diseases, detected through the aforementioned keyphrase-based system. More particularly, a set of 19 rare diseases is selected, which are reported annually to the National Registry of Rare Diseases (“Registro estatal de enfermedades Raras” or ReeR) or are in the process of being incorporated into this registry. The restriction of this study to the detailed analysis of 19 diseases is hence due to the existence of a standardized workflow for these diseases, which includes a specific disease profile or record for each one, defined at the national level^26^. The experts annotate the texts in which a rare disease has been detected based on the information they have about the patient and using nationally agreed-upon criteria, as these cases, once confirmed, are submitted to the national registry. In this way, an occurrence of the disease can be annotated as “confirmed”, “possible” but still with insufficient documentation to be confirmed, or “carrier of the disease” in some genetic diseases (this could be considered a confirmed case without clinical manifestation). On the other hand, the disease can also be annotated as “ruled out”, “ruled out in the person but present in the family history”, and “no information”. For the purposes of this work, the first three options are considered positive cases of the disease, while the last three annotations are considered negative cases.

With this information, we are able to construct a consolidated and definitive dataset on which subsequent experiments can be conducted for the detection and classification of rare diseases. To do this, all detected cases of the 19 considered diseases are taken and all the textual information of each patient is grouped into a single document. Considering that there are very few cases where the same patient presents more than one rare disease, only one disease per patient will be considered. Finally, in order to have both positive cases (suffering from one of the considered rare diseases) and negative cases (that do not present any of the considered diseases), a “None” label will be created. Both the cases for which our system detected a rare disease but the experts ruled it out, and cases where the disease indicated by the experts is different from the one detected and also lies outside the ReeR registry are labeled as “None”.

**Fig. 2:**
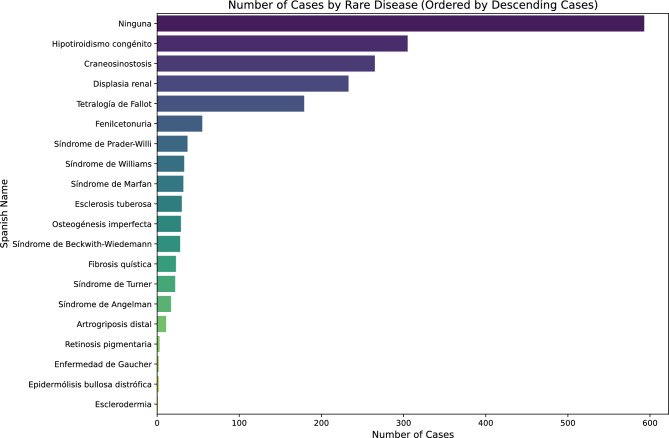
Number of cases by rare disease, ordered by descending cases.

The total number of cases in the consolidated dataset is 1,900. Table 6 shows the names in Spanish and English of the 20 possible labels in the dataset (19 rare diseases and label “None”), together with the total number of cases for each label. The label distribution is also shown in Figure 2 for better understanding.

**Table 6:** Statistics of the final dataset.

Spanish name	English name	Number of cases
Ninguna	None	593
Artrogriposis distal	Distal arthrogryposis	11
Craneosinostosis	Craniosynostosis	265
Displasia renal	Renal dysplasia	233
Enfermedad de Gaucher	Gaucher disease	2
Epidermólisis bullosa distrófica	Dystrophic epidermolysis bullosa	2
Esclerodermia	Scleroderma	1
Esclerosis tuberosa	Tuberous sclerosis	30
Fenilcetonuria	Phenylketonuria	55
Fibrosis quística	Cystic fibrosis	23
Hipotiroidismo congénito	Congenital hypothyroidism	305
Osteogénesis imperfecta	Osteogenesis imperfecta	29
Retinosis pigmentaria	Retinitis pigmentosa	3
Síndrome de Angelman	Angelman syndrome	17
Síndrome de Beckwith-Wiedemann	Beckwith-Wiedemann syndrome	28
Síndrome de Marfan	Marfan syndrome	32
Síndrome de Prader-Willi	Prader-Willi syndrome	37
Síndrome de Turner	Turner syndrome	22
Síndrome de Williams	Williams syndrome	33
Tetralogía de Fallot	Tetralogy of fallot	179
Total		1,900

Both the table and the figure clearly show the significant class imbalance present in the dataset. This is a crucial aspect regarding further experiments and evaluation of the obtained results, since those classes presenting a very low number of instances in the dataset are less likely to be correctly classified by a supervised algorithm. However, this issue will be taken into account when comparing different systems.

**Fig. 3:**
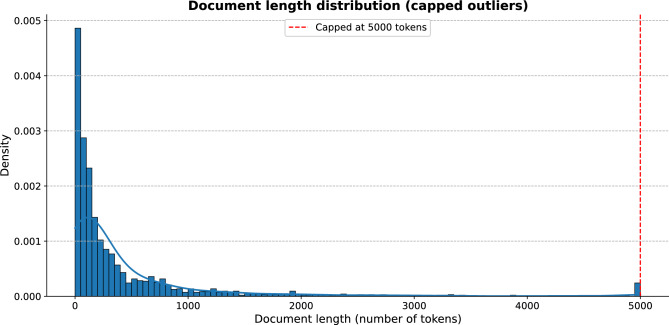
Histogram with the length distribution of the clinical notes in the consolidated dataset. All documents with more than 5,000 tokens are represented within the same bin (right side of the chart).

Figure 3 shows the length distribution (in number of tokens) for the clinical notes that compose the consolidated dataset.

As it can be seen in the figure, most of the documents in the dataset contain less than 1,000 tokens. On the other hand, there are 23 clinical notes with more than 5,000 tokens, which have been grouped within the last bin in the chart, in order to make the graph easier to interpret.

Regarding the ICPC-2 codes corresponding to congenital malformations related to patients in the final dataset, the most common codes associated to patients suffering from each of the selected rare diseases, together with the percentage of occurrence over the total number of patients for each disease, can be found in Appendix B (Table B1).

### Rare disease classification

Once the detection of the proposed subset of rare diseases has been performed and validated, a classification task can be designed in a way that a system receiving a text corresponding to the patient’s medical history must determine whether the patient suffers from any of the rare diseases considered for the task, or there is no evidence in the text of the patient being affected by any of them.

In this section, the various systems employed to address the problem of rare disease classification are proposed and described. The dataset generated in “Dataset creation”’ will be used for the task, and both a semi-supervised system and several supervised systems based on language models will be tested, with the aim of comparing their performance.

#### Semi-supervised system

The system based on the use of keyphrases described in “Keyphrase-based semi-supervised rare disease detection” is employed for performing rare disease classification on the final dataset. It is important to consider that, although this system was used for generating the initial detection of rare diseases on the initial corpus, it is also prone to make mistakes (both false positives and false negative). In this case, as it is mentioned in “Dataset creation”, most of the instances annotated as “None” in the dataset come from cases for which the keyphrase-based system detected a particular disease within the subset considered by the experts, but then those experts ruled out this disease in that particular patient. Those cases will become false positives for the keyphrase-based system regarding the consolidated dataset. On the other hand, there are also several cases for which the experts annotated some instances as mentioning a particular rare disease, while this disease had not been detected by the keyphrase-based system. Those will become false negatives for the keyphrase-based system in the rare disease classification task.

Given the semi-supervised nature of the keyphrase-based system, this system can be evaluated on the entire dataset of 1900 instances without the need to separate it into training and test subsets.

#### Pre-trained systems

With the aim of fully exploiting the generated rare disease classification dataset and exploring all the possibilities offered by the current state of the art for performing classification on biomedical texts, a series of additional techniques are proposed to address the problem. Specifically, we focus on discriminative language models and prompt-based generative large language models (LLMs) to explore their performance in this task. To this end, two pre-trained language models in Spanish with biomedical texts will be selected, both of them based on the Transformer architecture^27^: on one hand, a RoBERTa model^28^, pre-trained with Spanish clinical and biomedical information from various sources^29^ is explored. The main limitation of this model is the maximum document size it can process, which is 512 tokens. To address this issue, a Longformer model^30^, also pre-trained in Spanish^31^ with the same type of texts as the RoBERTa model, is also studied. This model allows for documents up to 4,096 tokens long, this way being able to better process the documents in the proposed dataset. Fine-tuning will be performed on both models to adapt them to the problem at hand. Additional information regarding hyperparameters, hardware specifications and training/inference times can be found in Appendix C.

In addition to exploring these supervised discriminative models, we are also interested in exploring the latest instruction-based generative models. In this regard, we have selected Llama 3^32^, one of the latest open-source models published. Both Llama 3 and its previous versions have shown very good performance in a variety of biomedical tasks^33,34^. Specifically, we will use the “Llama 3 8B” model, with 8 billion parameters. It is important to remark that Llama 3 is a large language model trained in texts written mainly in the English language. Although Spanish is likely to be among the languages the model has handled during its extensive training process, it is not its primary language, so the system’s performance will probably not reach the results achievable with models specifically trained in Spanish. Two different configurations of this model will be tested: the first will be a ’zero-shot’ methodology, in which the text to be classified is provided to the model along with a prompt indicating the possible values that can be selected for classification (the 19 rare diseases considered, plus the ’None’ label if no rare disease is detected in the text). In this case, no training or fine-tuning of the model is performed, hence it can be evaluated on the whole dataset in a similar way to the methodology followed with the semi-supervised keyphrase-based system. In the second experiment with Llama, instruction tuning will be performed on the model using the Low Rank Adaptation (LoRA) technique^35^. This technique aims at reducing the computational cost of training large models by decomposing the neural network’s weight matrix into two lower-dimensional matrices whose training implies less computational effort. As mentioned before, additional information about the technical specifications of the models employed can be found in Appendix C. Moreover, details of the prompt employed for generating the Llama 3 classification for each clinical note can be found in Appendix D (Figure D1). The prompt shown in the Appendix was used both in the zero-shot Llama 3 system and in the fine-tuned Llama 3 system. In the former case, the prompt is used to ask the model to generate the most likely rare disease label associated with the given clinical note (or the label “None” if no disease is found). In the second case, during the training phase, the prompt is accompanied by the correct label, hence following an instruction tuning methodology. During the testing phase, however, only the prompt is provided to the model, which is then asked to generate the predicted label.

It is important to remark that, although the zero-shot Llama model does not require training on labeled data, we have included it alongside the fine-tuned supervised models (RoBERTa, Longformer and fine-tuned LLaMA 3) to facilitate comparison between different uses of pre-trained models and LLMs under a common evaluation framework.

In all cases where a training process is necessary (RoBERTa and Longformer models, and Llama 3 model with LoRA-based fine-tuning), the same methodology will be followed: a stratified 5-fold cross-validation which maintains the existing class distribution in the train and test splits as much as possible. In this setting, each cross-validation fold uses 80% of the data for training and 20% for testing, ensuring that every instance in the dataset serves as part of a test set exactly once. After all five folds are evaluated, performance metrics are aggregated across the full set of 1,900 instances of the dataset, enabling direct comparison with the results obtained from the keyphrase-based and zero-shot Llama 3 systems. Within the 80% of the data dedicated to the training process, 10% is reserved as a validation split. In unsupervised learning^36^, the evaluation of a system often involves using the entire dataset without splitting it into training and test sets. This is because unsupervised (or semi-supervised in this case) methods do not optimize a model based on labeled outcomes, but rather aim to identify inherent structures or patterns in the data. By analyzing all the available data, the system can fully explore these structures and provide a more comprehensive evaluation of its performance. Furthermore, when no labeled ground truth exists, traditional train-test splits are unnecessary, as the focus shifts to metrics like clustering quality, reconstruction error, or similarity to known patterns. In addition, the small size of the corpus is also an important reason for evaluating the keyphrase-based system and the zero-shot Llama 3 system using the full dataset. This approach is hence consistent with methodologies reported in the literature, where, for unsupervised and semi-supervised tasks, the use of the full corpus allows capturing global relationships and maximizing the information available for evaluation. In contrast, for the supervised system, 5-fold stratified cross-validation was used to ensure a robust estimate of the generalization capabilities of the models.

In any case, the main objective is not necessarily to directly compare the behavior of a semi-supervised method with that of supervised ones, since the conditions under which they are evaluated are not exactly the same. Rather, synergy between the two types of systems can be analyzed through this methodology, and thus the appropriateness of applying one or the other depending on the data available.

No pre-processing steps are applied when using any of the pre-trained systems (RoBERTa, Longformer and Llama 3) for performing rare disease classification. In these cases, the original texts are employed, and tokenization is carried out using the respective tokenizers of each model as available in Huggingface [

Technical information including hardware specifications of the equipment employed for performing the experiments, as well as training and inference times of each model can be found in Appendix C.

## Results

In this section, the main results obtained by the different systems described in the previous section on the rare disease classification dataset will be presented. The primary metrics used for result analysis will be Precision, Recall, and F-Measure, and both micro and macro metrics will be studied. Macro metrics consider the overall performance of the models, giving equal importance to each of the classes (in this case, rare diseases) considered. On the other hand, micro metrics allow us to analyze the system’s behavior in more detail for each considered case. To this end, metrics associated with each of the rare diseases studied will be shown in order to compare the performance of each proposed model, taking into account the amount of available information for each disease. As mentioned in the previous section, all systems were evaluated on the same dataset of 1,900 clinical notes. The systems that do not require training on the dataset (the keyphrase-based system and the zero-shot Llama 3 model) were evaluated directly on the full dataset. For the supervised systems (RoBERTa, Longformer, and fine-tuned LLaMA 3), a 5-fold cross-validation procedure was conducted, ensuring that each instance in the dataset was used as a test sample exactly once. The aggregated results from the five folds therefore represent an evaluation over the full set of 1,900 instances, and are thus comparable to those obtained by the keyphrase-based and zero-shot Llama 3 systems.

Table 7 shows results obtained by the keyphrase-based system described in “Keyphrase-based semi-supervised rare disease detection” for each of the 19 rare diseases within the ReeR registry and the “None” class. Precision, Recall and F-Measure are computed for each possible label, and micro-average and macro-average metrics are also shown. Then, Table 8 shows the same metrics for the supervised systems based on RoBERTa and Longformer models, and Table 9 presents the metrics for the systems based on the Llama 3 model (both zero-shot and LoRA-based fine-tuned).

**Table 7:** Results obtained by the semi-supervised keyphrase-based system. Bold indicate that the model is offering the best result for a particular rare disease across all tested models. The whole dataset was employed for the evaluation of this system.

Rare disease	Precision	Recall	F-measure
Ninguna (*none*)	0.4883	0.6661	0.5635
Artrogriposis distal	0.8571	0.5455	**0.6667**
Craneosinostosis	0.6434	0.9736	0.7748
Displasia renal	0.8981	0.6052	0.7231
Enfermedad de Gaucher	1.0000	0.5000	**0.6667**
Epidermólisis bullosa distrófica	1.0000	1.0000	**1.0000**
Esclerodermia	0.5000	1.0000	0.6667
Esclerosis tuberosa	0.7879	0.8667	**0.8254**
Fenilcetonuria	0.9615	0.4545	0.6173
Fibrosis quística	0.4444	0.1739	**0.2500**
Hipotiroidismo congénito	0.9000	0.6197	0.7340
Osteogénesis imperfecta	0.9474	0.6207	0.7500
Retinosis pigmentaria	0.5000	0.3333	**0.4000**
Síndrome de Angelman	1.0000	0.4706	**0.6400**
Síndrome de Beckwith-Wiedemann	0.9000	0.3214	0.4737
Síndrome de Marfan	0.7692	0.3125	0.4444
Síndrome de Prader-Willi	0.9091	0.2703	0.4167
Síndrome de Turner	0.9167	1.0000	**0.9565**
Síndrome de Williams	0.9444	0.5152	0.6667
Tetralogía de Fallot	0.9928	0.7654	0.8644
**Micro-average**	**0.6737**	**0.6737**	**0.6737**
**Macro-average**	**0.8180**	**0.6007**	**0.6927**

**Table 8:** Results obtained by the RoBERTa-based and Longformer-based systems. Bold indicate that the model is offering the best result for a particular rare disease across all tested models. 5-fold Cross-Validation was employed for the evaluation of these systems.

	RoBERTa			Longformer		
Rare disease	Precision	Recall	F-measure	Precision	Recall	F-measure
Ninguna (*none*)	0.6447	0.7926	0.7110	0.6621	0.8128	**0.7298**
Artrogriposis distal	0.0000	0.0000	0.0000	0.0000	0.0000	0.0000
Craneosinostosis	0.8130	0.8038	0.8083	0.8182	0.8151	**0.8166**
Displasia renal	0.8261	0.8155	0.8207	0.8270	0.8412	**0.8340**
Enfermedad de Gaucher	0.0000	0.0000	0.0000	0.0000	0.0000	0.0000
Epidermólisis bullosa distrófica	0.0000	0.0000	0.0000	0.0000	0.0000	0.0000
Esclerodermia	0.0000	0.0000	0.0000	0.0000	0.0000	0.0000
Esclerosis tuberosa	0.8500	0.5667	0.6800	0.7778	0.7000	0.7368
Fenilcetonuria	0.8298	0.7091	**0.7647**	0.8810	0.6727	0.7629
Fibrosis quística	0.0000	0.0000	0.0000	0.0000	0.0000	0.0000
Hipotiroidismo congénito	0.9122	0.8852	**0.8985**	0.9236	0.8721	0.8971
Osteogénesis imperfecta	0.9091	0.6897	0.7843	0.9091	0.6897	0.7843
Retinosis pigmentaria	0.0000	0.0000	0.0000	0.0000	0.0000	0.0000
Síndrome de Angelman	1.0000	0.0588	0.1111	0.0000	0.0000	0.0000
Síndrome de Beckwith-Wiedemann	0.6957	0.5714	0.6275	0.9000	0.6429	**0.7500**
Síndrome de Marfan	0.9000	0.5625	**0.6923**	0.8947	0.5313	0.6667
Síndrome de Prader-Willi	0.6400	0.4324	0.5161	0.7500	0.5676	0.6462
Síndrome de Turner	0.9524	0.9091	0.9302	0.9130	0.9545	0.9333
Síndrome de Williams	0.9444	0.5152	0.6667	0.7419	0.6970	**0.7188**
Tetralogía de Fallot	0.8710	0.9050	0.8877	0.9240	0.8827	**0.9029**
**Micro-average**	**0.7732**	**0.7732**	**0.7732**	**0.7874**	**0.7874**	**0.7874**
**Macro-average**	**0.5894**	**0.4608**	**0.5173**	**0.5461**	**0.4840**	**0.5132**

**Table 9:** Results obtained by the Llama (zero shot and LoRA-based fine-tuned) systems. Bold indicate that the model is offering the best result for a particular rare disease across all tested models. 5-fold cross-validation was employed for the evaluation of the fine-tuned Llama 3 system, while the zero-shot system was tested in an unsupervised manner on all the instances of the consolidated dataset.

	Llama 3 (zero-shot)			Llama 3 (fine-tuned)		
Rare disease	Precision	Recall	F-measure	Precision	Recall	F-measure
Ninguna (*none*)	0.3992	0.3339	0.3636	0.4972	0.9106	0.6432
Artrogriposis distal	0.5000	0.1818	0.2667	0.5000	0.1818	0.2667
Craneosinostosis	0.4101	0.5849	0.4821	0.8670	0.6151	0.7196
Displasia renal	0.2198	0.4850	0.3025	0.8968	0.4850	0.6295
Enfermedad de Gaucher	0.0000	0.0000	0.0000	0.0000	0.0000	0.0000
Epidermólisis bullosa distrófica	0.0000	0.0000	0.0000	0.3333	0.5000	0.4000
Esclerodermia	0.0000	0.0000	0.0000	1.0000	1.0000	**1.0000**
Esclerosis tuberosa	0.7500	0.4000	0.5217	0.8000	0.4000	0.5333
Fenilcetonuria	0.8636	0.3455	0.4935	1.0000	0.3455	0.5135
Fibrosis quística	0.4000	0.0870	0.1429	0.6667	0.0870	0.1538
Hipotiroidismo congénito	0.8087	0.3049	0.4429	0.9383	0.6984	0.8008
Osteogénesis imperfecta	0.8824	0.5172	0.6522	1.0000	0.6552	**0.7917**
Retinosis pigmentaria	0.0000	0.0000	0.0000	0.0000	0.0000	0.0000
Síndrome de Angelman	0.6154	0.4706	0.5333	1.0000	0.2353	0.3810
Síndrome de Beckwith-Wiedemann	0.8333	0.1786	0.2941	1.0000	0.4286	0.6000
Síndrome de Marfan	0.9412	0.5000	0.6531	0.9286	0.4063	0.5652
Síndrome de Prader-Willi	1.0000	0.1622	0.2791	1.0000	0.4865	**0.6545**
Síndrome de Turner	0.0714	0.5000	0.1250	0.8333	0.2273	0.3571
Síndrome de Williams	0.1194	0.2424	0.1600	0.9500	0.5758	0.7170
Tetralogía de Fallot	0.9857	0.3855	0.5542	0.9926	0.7486	0.8535
**Micro-average**	**0.3853**	**0.3853**	**0.3853**	**0.6789**	**0.6789**	**0.6789**
**Macro-average**	**0.4900**	**0.2840**	**0.3596**	**0.7602**	**0.4493**	**0.5648**

Regarding overall results, the keyphrase-based system is able to obtain the best scores for macro-average metrics. In general, these results are logical considering that the keyphrase-based system, based on the use of the dictionary of rare diseases and their variants, can classify diseases regardless of the number of cases available in the dataset. When it comes to supervised systems, the number of instances seen during training significantly conditions their ability to generalize and thus classify these underrepresented diseases in the test subset. All the diseases that are especially difficult to be classified by the RoBERTa and Longformer models (distal arthrogyposis, Gaucher Disease, dystrophic epidermolysis bullosa, scleroderma, cystic fibrosis, retinitis pigmentosa and Angelman Syndrome) contain less than 25 cases in the dataset, hence this value can be seen as a threshold to be exceeded when building datasets for rare disease classification. Models based on Llama 3 somehow lie between these two scenarios, as their ability to generalize depends more on the pre-training undergone by the model, which is based on a much larger amount of data than the RoBERTa and Longformer models. Additionally, these models are directly provided with the possible classes they can select in the classification through the input prompt. This is why even with a zero-shot approach, the models based on Llama 3 are able to offer classifications for almost all the diseases in the dataset. In particular, there are only four rare diseases for which the Llama 3 model with zero-shot methodology is not able to offer any correct classification, and only two diseases in the case of the fine-tuned Llama 3 model. This indicates that, beyond specific results, generative models represent an interesting research path to be explored for this particular task.

Regarding micro-average metrics, it is clear that those models which are closer to the language and the particular domain employed in the dataset, such as RoBERTa and Longformer, are able to offer better results than both the keyphrase-based system and the more generalistic Llama 3 models. This is probably due to the fact that their pre-training and training processes are much more specific, especially for those diseases with enough training data, hence their learning and generalization abilities are successfully exploited. In particular, there are 9 classes (8 rare diseases and class “None”) for which any of the BERT-based models (RoBERTa and Longformer) are able to offer the best F-Measure metrics, compared to 8 diseases for which the keyphrase-based system obtains better F-Measure and 3 diseases better classified by the fine-tuned Llama 3 model. This leads to an improvement of 11.37% in micro-average F-Measure of the best BERT-based model (Longformer) compared to the keyphrase-based system. On the other hand, the fine-tuned Llama 3 model obtains very similar micro-average values than the keyphrase-based system, overcoming it by only 0,52%.

**Fig. 4:**
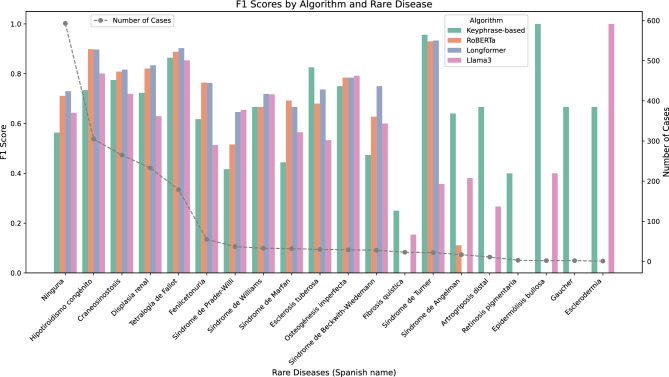
F-measure obtained by each of the developed systems (keyphrase-based, RoBERTa, Longformer and Llama 3 fine-tuned) for each rare disease in the dataset. Grey dashed line indicates the number of cases by rare disease. Diseases ordered (left to right) by descending number of cases.

Figure 4 provides a comparative illustration of the results obtained by the different systems developed in this work and places them in context with the characteristics of the dataset. Specifically, for each class (19 rare diseases plus the ’None’ class), the micro-average F-Measure values are presented for the keyphrase-based system, the two systems based on BERT architectures (RoBERTa and Longformer), and the fine-tuned Llama 3 system. The values for the zero-shot version of Llama 3 are not included, as it does not outperform the other systems for any of the dataset’s possible classes. The total number of instances for each class in the dataset is also shown as a grey dotted line, and diseases are ordered from left to right in the chart, according to this number of cases (descending order).

The figure clearly shows the dynamics followed by the different systems proposed in this work, with three distinct behaviors observable: when the number of instances available for a specific class is sufficiently high (more than approximately 40 instances), the systems based on fine-tuning a pre-trained model such as RoBERTa or Longformer achieve the best results, with Longformer performing the best on average between the two. This corresponds to the first six classes (from “Ninguna” (None) to “Fenilcetonuria”, inclusive). In all these cases, the keyphrase-based system and the fine-tuned Llama 3 system fall behind the other two techniques. This indicates that the availability of a good amount of training data benefits systems based on BERT architectures, even when the number of available instances is not excessively high.

A second differentiated behavior would be illustrated by those cases with around 20 to 40 instances per class, represented by the next six classes in the graph (from “Síndrome de Prader-Willi” to “Síndrome de Beckwith-Wiedemann”, inclusive). In these cases, there is much higher variability in the behavior of the different systems tested, with some diseases still showing better results for the BERT-based systems, but also diseases where the keyphrase-based system and even the Llama 3-based system outperform the other two.

Finally, as we move to the far right side of the graph, where the number of available instances for each class drastically decreases, only the keyphrase-based system and the Llama 3-based system offer acceptable results, with some cases where the BERT-based systems fail to classify any instances correctly.

These results, as previously mentioned, reinforce the idea that both the keyphrase-based system and generative systems like Llama 3 handle data scarcity much better than BERT-based systems. In the case of the keyphrase-based system, this is because it does not rely on training data but rather on its pattern-matching-based detection rules. For generative systems, they benefit from being pre-trained on vast amounts of data, which, combined with the fine-tuning that can be applied, allows them to correctly classify these underrepresented classes in the dataset.

The rare diseases that are best classified by each of the tested models, in terms of F-Measure, are as follows:

- Keyphrase-based model: distal arthrogryposis, Gaucher Disease, dystrophic epidermolysis bullosa, tuberous sclerosis, cystic fibrosis, retinitis pigmentosa, Angelman Syndrome and Turner Syndrome.
- RoBERTa model: phenylketonuria, congenital hypothyroidism and Marfan Syndrome.
- Longformer model: craniosynostosis, renal dysplasia, Beckwith-Wiedemann Syndrome, Williams Syndrome and tetralogy of Fallot.
- Llama 3 model (fine-tuned): scleroderma, osteogenesis imperfecta and Prader-Willi Syndrome.

Additionally, the Longformer model offers the best F-Measure in classifying the “None” label. Finally, the zero-shot Llama 3 model is not able to overcome the other models for any of the considered diseases.

## Discussion

In this section, the predictions generated by the various systems tested will be discussed. For this purpose, different scenarios encountered in the analysis of specific instances from the evaluation dataset will be presented, showing the text of the analyzed instance and studying the reasons that lead to correct detection by some models and incorrect detection by others.

Table 10 shows a collection of selected cases for which all the possible classification scenarios are covered.

**Table 10:** Case analysis. Bold text within column “evidence” indicates the likely textual evidence in each text fragment, in cases where this evidence appears.

Case no.	Evidence	Gold standard	Keyphrase-based	BERT-based	Llama 3 (fine-tuned)
1	“**Esclerosis tuberosa** comprobada por genética. Cojera. Exp: normal. Everolimus 5 mg.” (“*Tuberous sclerosis confirmed by genetics. Limping. Exp: normal. Everolimus 5 mg.*”)	Esclerosis tuberosa	Esclerosis tuberosa	Esclerosis tuberosa	Esclerosis tuberosa
2	“[...] está siendo estudiado por AF de embarazo interrumpido a la 15 sem por **Tetralogia de Fallot** [...] ” (“*[...] being studied due to a family history of interrupted pregnancy at 15 weeks due to Tetralogy of Fallot [...]*”)	Ninguna (*None*)	Tetralogía de Fallot	Tetralogía de Fallot	Tetralogía de Fallot
3	“No deformidad craneal congénita y desarrollo psicomotor normal. Aún así derivo para despistaje de **craneosinostosis**.” (“*No congenital cranial deformity and normal psychomotor development. Nevertheless, I refer for craniosynostosis screening.*”)	Craneosinostosis	Craneosinostosis	Ninguna (*None*)	Ninguna (*None*)
4	“En tratamiento con eutirox.” (“*On treatment with Euthyrox.*”)	Hipotiroidismo congénito	Ninguna (*None*)	Hipotiroidismo congénito	Hipotiroidismo congénito
5	“Se aprecian todas las suturas craneales abiertas asi como fontanela anterior, descartando craneosinostosis.” (“*All cranial sutures are open, as well as the anterior fontanelle, ruling out craniosynostosis.*”)	Ninguna (*None*)	Craneosinostosis	Ninguna (*None*)	Ninguna (*None*)
6	“[...] resuelvo. **Sd Marfan** y conviviente vulnerable.	Síndrome de Marfan	Ninguna (*None*)	Síndrome de Marfan	Síndrome de Marfan
7	“23 en cariotipo compatible con **S Williams**.”	Síndrome de Williams	Ninguna (*None*)	Síndrome de Williams	Síndrome de Williams
8	“Diagnostico postnatal de **T. Fallot**”	Tetralogía de Fallot	Ninguna (*None*)	Tetralogía de Fallot	Tetralogía de Fallot
9	“**Tratalogia de fallot** pendiente de cirugia”)	Tetralogía de Fallot	Ninguna (*None*)	Tetralogía de Fallot	Tetralogía de Fallot
10	“Sospechan **esclerosis tuberosa** no confirmada por derma ni RM; han aumentado dosis de depakine.” (“*They suspect tuberous sclerosis, not confirmed by dermatology or MRI; they have increased the dose of Depakine.*”)	Ninguna (*None*)	Esclerosis tuberosa	Ninguna (*None*)	Esclerosis tuberosa
11	“Gammagrafía tiroidea: tiroides ectópico sublingual. Levotroid 50: 1/2 / día. Hago receta de Eutirox 88.” (“*Thyroid scan: sublingual ectopic thyroid. Levotroid 50: 1/2 per day. I’ll prescribe Eutirox 88.*”)	Hipotiroidismo congénito	Ninguna (*None*)	Hipotiroidismo congénito	Ninguna (*None*)
12	“Pruebas metabólicas alteradas con sospecha de **hipotiroidismo congénito**. Se realiza nueva prueba del talón en el centro de salud. Pruebas metabólicas normales.” (“*Altered metabolic tests with suspected congenital hypothyroidism. A new heel prick test is performed at the health center. Metabolic tests are normal.*”)	Ninguna (*None*)	Hipotiroidismo congénito	Hipotiroidismo congénito	Ninguna (*None*)
13	“prematuridad, 31 semanas eg dg. al alta **sd prader. willi** dap, precisa sonda nasogástrica, enf. membrana hialina leve. Se contacta con enfermera de enlace.”	Síndrome de Prader-Willi	Ninguna (*None*)	Ninguna (*None*)	Síndrome de Prader-Willi

The table shows excerpts from the patient’s medical history text in column “Evidence”, selected based on the appearance of certain textual evidence that could lead to the detection of the disease. “Gold Standard” column indicates the correct classification according to the experts, while the remaining columns display the prediction made by each of the explored systems. It is important to remark that, for the sake of clarity and readability of the tables, we will group the predictions of the RoBERTa and Longformer models. That is, the column ’BERT-based’ indicates the prediction done by these models, hence only those cases where both models offer the same prediction will be taken into account. Additionally, regarding Llama 3, we will only show predictions by the LoRA-based fine-tuned version.

- Case 1: The case shows a straightforward scenario where all tested models correctly classify the rare disease (tuberous sclerosis). It is clearly written in the text in its usual form, followed by the word “comprobada” (“*confirmed*”), allowing both the keyphrase-based system and the models based on BERT and Llama 3 to detect it without any problem.
- Case 2: The case refers to a scenario where none of the analyzed models are able to correctly classify the disease. Specifically, in this case, they fail to select the “None” label. The text refers to a previous pregnancy of the mother that did not come to term due to the presence of Tetralogy of Fallot in the fetus. All the models detect the mention of the disease, but none of them have the ability to interpret that the mention refers to a family history antecedent, instead of to the current patient.
- Case 3: In this case, only the keyphrase-based system is able to correctly classify the disease (craniosynostosis). The correct classification shown by the Gold Standard is likely due to additional information that is not available in the text, since the sentence shown in the Table intuitively suggests that the health professional is only suspecting the disease, which is why the supervised methods dismiss it. However, since the mention of the disease is explicit, the keyphrase-based method still detects it and ultimately is the only one that gets it right.
- Case 4: From this case onward, scenarios where supervised models outperform the unsupervised technique are analyzed. In this particular case, the keyphrase-based system fails in detecting the disease because it is not mentioned in the text. However, the reference to the medication “Eutirox”, widely used in cases of hypothyroidism, allows all supervised models, which manage prior information (from either pre-training or fine-tuning), to correctly detect the disease (congenital hypothyroidism).
- Case 5: This case is a clear example of the need to correctly detect linguistic phenomena such as negation for performing an accurate classification. In the text, it is evident that the disease is ruled out. However, the negation detection module in the keyphrase-based model does not work properly for this instance, causing the system to detect the mention of craniosynostosis as positive. Supervised models, which are much more sophisticated, incorporate this knowledge about negation into their background knowledge and are capable of correctly classifying the label “None”.
- Cases 6 to 9: All these cases can be grouped into a scenario that clearly shows why supervised models add value to the classification of rare diseases compared to semi-supervised models such as the keyphrase-based system employed in this work. They are all cases where mentions of diseases are written in a particular manner, for instance with acronyms (“Sd Marfan”, “S Williams”, “T. Fallot”), or directly with spelling errors (“tratalogía” instead of “tetralogía”). In all these cases, the background knowledge handled by supervised language models allows them to correctly detect the disease despite these variations. On the other hand, the keyphrase-based model, lacking these variants in its dictionary, is unable to detect them. The English translation is not provided in these cases since the writing variations only make sense in Spanish.
- Cases 10 and 11: In these two cases, the keyphrase-based system fails to classify the disease, and among the supervised systems, only those based on BERT are able to detect it correctly. Generally, these are more subtle cases with additional information has been used for performing the classification in the Gold Standard, other than the textual content. In case 10, there is only suspicion of the disease mentioned; due to this explicit mention, the keyphrase-based model detects it as positive, as does the Llama 3 model. However, the correct classification is “None”, a label correctly predicted by the RoBERTa and Longformer-based models. In case 11, there is no mention of the disease, so the keyphrase-based system does not detect it. However, congenital hypothyroidism could be inferred from the tests and medications mentioned, similar to what happened in case 5. In this case, only the BERT-based models are capable of making this detection.
- Cases 12 and 13: Similarly to cases 10 and 11, these are more subtle examples in which only the Llama 3 model is able to correctly classify the rare disease, while the keyphrase-based and BERT-based models fail. In case 12, first information about the patient indicate the possibility of congenital hypothiroidism, while subsequent medical tests rule it out. Only Llama 3 is able to interpret the complete chain of facts that leads to classifying the instance as “None”. Case 13 is also an example of linguistic variation, similar to cases 6 to 9. However, in this case only Llama 3 is able to infer the correct disease (Prader-Willi Syndrome) from the available text.

To complement this case analysis, Table 11 shows the different possible scenarios regarding the combination of outputs from the evaluated systems, along with the number of cases in which each possible combination occurs.

**Table 11:** Number of cases for each of the possible combinations of outputs from each evaluated system. Only instances with the same output from RoBERTa and Longformer are considered. First column relates the output combination to the cases previously explained. Columns 2, 3 and 4 indicate whether the system predicts the correct (✓) or an incorrect (✗) label. Last column indicates the number of cases for each output combination. Last two rows correspond to combinations not explicitly described in the case analysis.

Example case	Keyphrase-based	BERT-based	Llama 3	No. cases
1	✓	✓	✓	832
2	✗	✗	✗	168
3	✓	✗	✗	41
4-9	✗	✓	✓	258
10-11	✗	✓	✗	58
12-13	✗	✗	✓	52
Not shown	✓	✗	✓	29
Not shown	✓	✓	✗	239

As indicated in the caption and similarly to the case analysis, only those cases in which the two BERT-based systems (RoBERTa and Longformer) offer the same prediction for an instance are considered. In addition, there are two scenarios (last two rows of the table) that have not been explicitly illustrated in the case analysis.

These results directly align with the quantitative results shown in Tables 7, 8 and 9, together with the behavior illustrated in Figure 4: in most cases all systems are able to correctly classify the instance (first row), while the number of scenarios for which all systems misclassify the instance is not high (second row). This indicates that, in general, the proposed systems offer good overall results. Then, the third and fourth rows (cases for which the keyphrase-based system performs better and cases for which any supervised model performs better, respectively), illustrate how using more complex models leads to better results in general. Although the fifth and sixth rows may seem to offer a similar number of cases, the differences between BERT-based models and the fine-tuned Llama 3 model must be analysed disregarding the behavior of the keyphrase-based model, this is, considering the fifth and eight rows together, and the sixth and seventh rows together. This way, the table shows how using any of the BERT-based models is a better choice in many more cases than employing the fine-tuned Llama 3 model for addressing the task.

## Conclusions

In this article, a complete pipeline oriented towards the detection and classification of rare diseases in pediatric clinical notes written in Spanish has been presented. Starting with a cohort of patients, a semi-supervised keyphrase-based system was applied to perform an initial detection of a specific set of rare diseases. Some linguistic aspects such as negation detection and mentions of rare diseases related to family members instead of the patient were taken into account in order to reduce the number of false positives. This initial detection was refined and validated by experts in the field, resulting in a consolidated dataset of rare diseases on which further classification experiments were conducted. Various models, both semi-supervised and supervised, have been proposed for the classification of rare diseases.

The obtained results reinforce the idea that there is a critical need for obtaining substantial amounts of high-quality data, so that the possibilities offered by supervised techniques, especially those based on large language models and generative artificial intelligence, can be fully exploited. Even with the characteristics of the developed dataset, which has a modest size and includes certain diseases with very few cases, these models are capable of delivering promising results in the classification of rare diseases, overcoming the proposed keyphrase-based model in terms of micro-average metrics, and obtaining promising scores for macro-average metrics. However, it is also important not to lose sight of the capabilities offered by unsupervised and semi-supervised models in dealing with these issues of limited information and data.

An additional analysis has been performed on individual cases for which the explored systems perform differently. In this analysis, the strengths provided by supervised systems (both those based on RoBERTa and Longformer, and those using generative models like Llama 3) become very evident, while the limitations of the keyphrase-based system are clearly shown. Therefore, the exploration of systems with deeper domain knowledge becomes highly necessary in such tasks, enabling them to make inferences that are difficult to achieve with other techniques.

### Limitations

The following limitations have been identified throughout the development of the work presented in this paper, in relation to various aspects of the conducted research:

- Data availability and dataset size: as discussed throughout this paper, the limited availability of data is one of the fundamental challenges in detecting and classifying rare diseases. Obtaining clinical notes, medical reports, and texts from the biomedical domain, even when they are not annotated, is not straightforward, especially when working with languages other than English. This information gathering requires highly detailed collaboration agreements with medical institutions such as hospitals, regional and national registries or health departments, among others. Furthermore, annotating this type of corpus is also very costly, as it requires the involvement of domain experts with deep knowledge of the subject matter and cannot rely on more agile annotation methods used for other types of datasets, such as crowdsourcing and collaborative annotation. For this reasons, the consolidated dataset, in its current version, is still very limited, and thus the generalization and extrapolation of the obtained results must be approached with caution.
- Dataset publishability: another major challenge with this type of dataset, related to the issues mentioned above, is the difficulty of making it publicly accessible to the research community, thereby allowing other researchers to contribute to scientific progress on the topic. Patient medical information is extremely sensitive, and in this case, it involves pediatric patients, i.e., minors. The processes of anonymization, review by ethical committees, and other necessary actions to ensure the possibility of publishing such a dataset are slow and costly.
- Model explainability: one of the most significant challenges associated with the use of deep learning models is their explainability. In the biomedical domain, the ability to provide detailed explanations of the decisions and predictions made by an automated system is crucial for its potential implementation and use. However, in many cases, these systems act as black boxes, making their outputs difficult to interpret. Although generative models have made progress in this area by enabling natural language explanations for their decisions, it is important to continue researching this field to develop predictive models that can be effectively employed in medical practice.

### Future work

All the previous conclusions extracted from the research, together with the limitations envisioned during its development, help us to depict the following future lines of research:

- Collection of new information: one of the main issues observed throughout this work, particularly concerning the use of supervised systems, is the lack of data associated with certain diseases, which results in a poorer performance of some of the supervised models explored. For this reason, efforts should be made to collect a larger amount of instances, especially for those diseases for which the current number of cases in the dataset is very low. As discussed in Section 4, some systems, especially those based on generative models, are able to start offering interesting results with not so many training instances. Therefore, even a small increase in the total number of cases related to a disease can lead to a significant improvement in the results obtained by these models for classifying that disease. However, when it comes to rare diseases, obtaining a significant number of cases will always be challenging (and the rarer the disease, the lower the availability of cases), which represents an important limitation for these processes.
- Generative AI: although the BERT-based models offer the best results in micro-average metrics, the Llama 3 (fine-tuned) model is the one that most closely approaches the performance of the keyphrase-based system in terms of macro-average metrics. Therefore, it is interesting to further investigate this type of generative models, experimenting with alternative models and focusing on fine-tuning and hyperparameter exploration. Improvements in macro-average metrics are expected to also result in improvements in micro-average metrics. In this regard, the application of few-shot learning techniques on generative models will also be explored in order to determine its appropriateness for the task, as well as its advantages regarding zero-shot and fine-tuning techniques.
- Hybrid models: as it can be clearly observed in the results, both the semi-supervised keyphrase-based model and the supervised models are capable of delivering good results for different subsets of diseases. For this reason, it would be interesting to explore the possibility of developing hybrid models that take advantage of the benefits each of these systems offers separately, in order to combine the best of both worlds. Previous works have already shown the potential of hybrid approaches for performing related tasks such as rare disease phenotyping^37^.
- Explainability: the application of explainability techniques such as SHAP (SHapley Additive exPlanation)^38^ or LIME (Locally Interpretable Model Agnostic Explanations)^39^ could be very useful for a better interpretation and understanding of the decisions taken by the supervised systems.

## Supplementary Information

Supplementary Information.

## Data Availability

The data that support the findings of this study are not openly available due to reasons of sensitivity, as they contain information that could compromise the privacy of the patients populating the original data cohort. The corresponding author may be contacted to discuss reasonable requests for data access, subject to appropriate ethical and institutional approvals.
