# Supplementary material for: An integrated approach for rare disease detection and classification in Spanish pediatric medical reports
Source: Sci Rep. 2025 Oct 30;15:37973. doi: 10.1038/s41598-025-21827-4 (PMC12575776; doi:10.1038/s41598-025-21827-4)
Supplement: Supplementary file 1 — Supplementary Information. [file 41598_2025_21827_MOESM1_ESM.pdf]

A ICPC-2 Codes and Descriptions

ICPC-2 code	Spanish description	English description
A90	Anomalías congénitas múltiples no especificadas	Congenital anomaly not otherwise specified/multiple
B79	Otras anomalías congénitas de sangre / órganos hematopoyéticos / linfáticos	Congenital anomaly blood/lymph other
D81	Anomalías congénitas del aparato digestivo	Congenital anomaly digestive system
F81	Otras anomalías oculares congénitas	Congenital anomaly eye other
H80	Anomalías congénitas del aparato auditivo	Congenital anomaly of ear
K73	Anomalías congénitas cardiovasculares	Congenital anomaly cardiovascular
L82	Anomalías congénitas del aparato locomotor	Congenital anomaly musculoskeletal
N85	Anomalías congénitas del sistema nervioso	Congenital anomaly neurological
R89	Anomalías congénitas del aparato respiratorio	Congenital anomaly respiratory
S83	Otras lesiones cutáneas congénitas	Congenital skin anomaly other
T80	Anomalías congénitas endocrinas/metabólicas	Congenital anomaly endocrine/metabolic
U85	Anomalías congénitas del aparato urinario	Congenital anomaly urinary tract
W76	Anomalías congénitas que complican el embarazo	Congenital anomaly complicating pregnancy
X83	Anomalías congénitas del aparato genital femenino	Congenital anomaly, female genital
Y84	Anomalías congénitas del aparato genital masculino	Congenital anomaly, male genital

Table A1. ICPC-2 Codes appearing in the initial data cohort employed in this study, and their descriptions. All of them refer to congenital malformations.

B Most common codes assigned to patients in the final dataset

Rare Disease (Spanish)	Rare Disease (English)	ICPC-2 codes
Artrogriposis distal	Distal arthrogryposis	L82 (90.91%)
Craneosinostosis	Craniosynostosis	L82 (92.83%)
Displasia renal	Renal dysplasia	U85 (90.56%)
Enfermedad de Gaucher	Gaucher disease	L82 (50%) / T80 (50%)
Epidermólisis bullosa distrófica	Dystrophic epidermolysis bullosa	S83 (100%)
Esclerodermia	Scleroderma	S83 (100%)
Esclerosis tuberosa	Tuberous sclerosis	A90 (80%)
Fenilcetonuria	Phenylketonuria	T80 (65.45%)
Fibrosis quística	Cystic fibrosis	K73 (30.43%) / D81 (26.09%) / L82 (26.09%)
Hipotiroidismo congénito	Congenital hypothyroidism	T80 (92.79%)
Osteogénesis imperfecta	Osteogenesis imperfecta	L82 (89.66%)
Retinosis pigmentaria	Retinitis pigmentosa	T80 (33.33%) / F81 (33.33%) / D81 (33.33%)
Síndrome de Angelman	Angelman Syndrome	A90 (76.47%)
Síndrome de Beckwith-Wiedemann	Beckwith-Wiedemann Syndrome	A90 (75%)
Síndrome de Marfan	Marfan Syndrome	A90 (62.5%)
Síndrome de Prader-Willi	Prader-Willi Syndrome	A90 (86.49%)
Síndrome de Turner	Turner Syndrome	A90 (100%)
Síndrome de Williams	Williams Syndrome	A90 (66.67%)
Tetralogía de Fallot	Tetralogy of Fallot	K73 (98.32%)

Table B1. Most assigned ICPC-2 codes per disease in the final dataset. Percentage over the total number of patients with the disease is shown in parentheses.

C Technical specifications

C.1 Hyperparameters

- **RoBERTa and Longformer models:** A batch size of 8 has been employed for training the RoBERTa model, reduced to 2 when it comes to the Longformer model. The total number of epochs is 10 in both cases and the best model of all the iterations is saved for inference. A learning rate of $2e^{-5}$ is used, and the selected optimizer is AdamW. Finally, categorical cross-entropy is used as the loss function for fine-tuning the models.

- **Fine-tuned Llama 3 model:** The model is trained for 3 epochs, with a batch size of 8, a learning rate of $2e^{-4}$, and a weight decay of 0.001. The optimizer is AdamW. As for the specific parameters of the LoRA technique, the default parameters of $alpha = 16$, $r = 64$, and $dropout = 0.1$ have been selected.

C.2 Hardware

All training and inference for the most complex models (RoBERTa, Longformer and Llama 3) have been done on Nvidia RTX A5000 GPUs (24 GB of RAM each). In particular, RoBERTa and Longformer experiments have been performed on 4 GPUs while, due to availability issues, all Llama 3 experiments (zero-shot and fine-tuning) have been conducted on a single GPU.

C.3 Training and inference times

- **RoBERTa**: An average of 104 seconds of training and 61 seconds of inference per cross-validation fold, for a total of 520 seconds of training and 305 seconds of inference (825 seconds or 13 minutes and 45 seconds).

- **Longformer**: An average of 1,475 seconds of training and 3,569 seconds of inference per cross-validation fold, for a total of 7,375 seconds of training and 17,845 seconds of inference (25,220 seconds or 7 hours and 20 seconds).

- **Zero-shot Llama 3**: Inference time of 1710 seconds for evaluating the whole dataset (28 minutes and 30 seconds).

- **Fine-tuned Llama 3**: An average of 6,748 seconds of training and 342 seconds of inference per cross-validation fold, for a total of 33,740 seconds of training and 1710 seconds of inference (35,450 seconds or 9 hours, 50 minutes and 50 seconds).

D Prompt used for the Llama 3 experiments

```
Analyze the text written in Spanish, enclosed in square brackets and determine
whether the text has any relationship whatsoever with any of the following
rare diseases: ‘craneosinostosis’, ‘hipotiroidismo congenito’, ‘patologia
renal’, ‘sindrome de marfan’, ‘esclerosis tuberosa’, ‘enfermedad de gaucher’,
‘fenilcetonuria’, ‘fibrosis quistica’, ‘osteogenesis imperfecta’, ‘retinosis
pigmentaria’, ‘sindrome de angelman’, ‘sindrome de wiedemann-beckwith’,
‘sindrome de willi-prader’, ‘sindrome de williams’, ‘tetralogia de fallot’,
‘esclerodermia’, ‘artrogriposis distal’, ‘epidermolisis bullosa distrofica’,
‘sindrome de turner’. Return the answer as the corresponding disease
label ‘craneosinostosis’, ‘hipotiroidismo congenito’, ‘patologia renal’,
‘sindrome de marfan’, ‘esclerosis tuberosa’, ‘enfermedad de gaucher’,
‘fenilcetonuria’, ‘fibrosis quistica’, ‘osteogenesis imperfecta’, ‘retinosis
pigmentaria’, ‘sindrome de angelman’, ‘sindrome de wiedemann-beckwith’,
‘sindrome de willi-prader’, ‘sindrome de williams’, ‘tetralogia de fallot’,
‘esclerodermia’, ‘artrogriposis distal’, ‘epidermolisis bullosa distrofica’,
’sindrome de turner’. If the text has no relationship to any of the proposed
diseases, return ‘ninguna’ or ‘none’.
```

Figure D1. Prompt used for Llama 3 response generation.
